# Knowledge and awareness of glucose-6-phosphate dehydrogenase deficiency in the Al-Baha region, Saudi Arabia: a cross-sectional study

**DOI:** 10.25122/jml-2025-0178

**Published:** 2026-09

**Authors:** Fahad Ali Alamr, Raghad Ahmed Alamri, Alanoud Saleh Alzahrani, Nada Saleh Algamdi, Ohood Mubarak Algamdi, Mashaer Rashed Alzhrani

**Affiliations:** 1 Department of Pediatrics, Faculty of Medicine, Al-Baha University, Al-Baha, Saudi Arabia; 2 Faculty of Medicine, Al-Baha University, Al-Baha, Saudi Arabia

**Keywords:** glucosephosphate dehydrogenase deficiency, health knowledge, attitudes, practice, anemia, hemolytic, Saudi Arabia, cross-sectional studies, surveys and questionnaires, CI, confidence interval, G6PD, glucose-6-phosphate dehydrogenase, OR, odds ratio, RBC, red blood cell, SAR, Saudi riyal, SPSS, Statistical Package for the Social Sciences

## Abstract

Glucose-6-phosphate dehydrogenase (G6PD) deficiency is a common inherited enzymopathy and a leading cause of hemolytic anemia, with a high prevalence in Saudi Arabia. Affected individuals are often asymptomatic until exposed to specific triggers, making public awareness essential for prevention. This study evaluated knowledge and awareness of G6PD deficiency among adults in the Al-Baha region. A cross-sectional survey was conducted between January and July 2025 among residents aged 18 years and older, using a structured online questionnaire covering general knowledge, symptom recognition, triggers, and management. Knowledge was scored across 33 items and grouped into three bands. We assessed associations with demographic variables using the chi-square test and multivariable logistic regression (*P* < 0.05). Of 487 participants, 298 (61.2%) were female and 472 (96.9%) Saudi nationals; 226 (46.4%) were aged 20–30 years. Although 311 (63.9%) had heard of the condition, the mean knowledge score was 6.22 ± 1.51 of 33, and 264 (54.2%) fell into the lowest band. Fava beans were correctly identified as a trigger by 450 respondents (92.4%), but awareness of infections (28.5%), aspirin (13.6%), and paracetamol (13.1%) was low. Knowledge level was associated with gender (*P* = 0.005) and age (*P* = 0.008); in the adjusted analysis, age was the only independent predictor, participants aged 31–40 years having lower odds of high knowledge than those aged 20–30 years (OR = 0.260; 95% CI, 0.114–0.591; *P* = 0.002). Public awareness of G6PD deficiency in Al-Baha is inadequate, particularly regarding non-dietary triggers, underscoring the need for targeted educational interventions.

## INTRODUCTION

Glucose-6-phosphate dehydrogenase (G6PD) deficiency is the most common enzyme disorder worldwide, affecting approximately 400 million individuals, particularly in regions where malaria is or was endemic [1]. It is an X-linked genetic disorder caused by mutations in the *G6PD* gene that result in amino acid substitutions, leading to enzyme instability and reduced activity [1,2]. G6PD catalyzes the first step of the pentose phosphate pathway, generating nicotinamide adenine dinucleotide phosphate, which is essential for maintaining reduced glutathione in red blood cells (RBCs). This process, together with catalase activity, protects RBCs from oxidative damage; enzyme deficiency therefore renders erythrocytes highly susceptible to oxidative stress and hemolysis [1,3].

Clinically, most individuals with G6PD deficiency remain asymptomatic until exposed to oxidative triggers such as infections, certain medications, or specific foods [4,5]. When hemolysis occurs, patients may present with fatigue, pallor, jaundice, dark urine, fever, and back pain; in neonates, G6PD deficiency is a major cause of jaundice [2,6]. Common precipitating factors include antimalarial drugs such as primaquine, certain antibiotics and analgesics, and natural substances such as henna [4,5]. Fava beans are the best-known dietary trigger, but other legumes, soya products, and tonic water may also induce oxidative stress and hemolysis in susceptible individuals [5].

Globally, G6PD deficiency is most prevalent in tropical Africa, the Middle East, and parts of Asia, with gene frequencies ranging from 5% to 25% [7]. In Saudi Arabia, the overall prevalence is approximately 9% in men and 4% in women, with regional variation—notably higher rates in the Eastern Province and lower rates in the Northern Province [8]. In the Al-Baha region, prevalence has been reported above the national average, at 12% in men and 11% in women [8]. Despite this, few studies have examined public knowledge and awareness of G6PD deficiency within the Kingdom.

Previous research has been conducted at national [9] and regional levels—including the Eastern Province [10], the Northern Region [11], Jazan [12], and Al-Qatif [13]—but no comprehensive data are available for Al-Baha. We hypothesized that public knowledge and awareness of G6PD deficiency in the Al-Baha region were limited, and that awareness levels varied with demographic characteristics. This study therefore aimed to address that gap by assessing knowledge and awareness of G6PD deficiency among the general population of the Al-Baha region.

## MATERIAL AND METHODS

### Study design and setting

A cross-sectional, observational, community-based study was conducted in the Al-Baha region of Saudi Arabia from January 2025 to July 2025 to assess knowledge and awareness of G6PD deficiency among the general population, using an online survey distributed within the region.

### Participants

The target population comprised all residents of the Al-Baha region aged 18 years and above who consented to participate. Individuals younger than 18 years, healthcare professionals, non-residents of the region, those submitting incomplete questionnaires, and those declining participation were excluded. Participants were recruited on a voluntary basis by convenience sampling through online distribution of the questionnaire.

### Sample size

The required sample size was determined using the Raosoft online sample size calculator, with a 95% confidence level, a 5% margin of error, and the estimated total population of the Al-Baha region. The minimum calculated sample size was 384 participants, which was increased to 422 to allow for a 10% non-response rate.

### Questionnaire

Data were collected through a structured online questionnaire developed from the instruments used in two previous studies, Hamali *et al*. [12] and Alharbi *et al*. [14]. The questionnaire was reviewed by a hematology consultant to ensure content relevance and clarity, and was pilot tested on a small group of participants to assess clarity, comprehension, and feasibility; feedback from the pilot was used to refine wording and structure before final distribution. The questionnaire was administered in Arabic.

The questionnaire comprised three sections. The first collected sociodemographic data, including gender, nationality, age, education level, marital status, and family income. The second assessed general knowledge of G6PD deficiency, and the third addressed knowledge of its triggers, symptoms, and management.

### Endpoints

The primary endpoint was the overall level of knowledge and awareness of G6PD deficiency among the adult population of the Al-Baha region. Secondary endpoints were knowledge across individual domains (general awareness, symptom recognition, triggers, and management) and the association between knowledge level and sociodemographic variables.

### Scoring

The knowledge questionnaire comprised 33 items across four domains: general awareness (11 items), symptom recognition (6 items), triggering factors (9 items), and management and preventive actions (7 items). Each correct response scored 1 point, and each incorrect or “don’t know” response scored 0, for a maximum possible score of 33.

Total scores were obtained by summing across all items. Because observed scores clustered within a narrow range relative to the maximum, knowledge was grouped into three bands based on the score distribution: low (score ≤6), medium (score 7), and high (score ≥8).

### Statistical analysis

Data were checked for completeness and accuracy before analysis. Statistical analysis was performed using SPSS version 26 (IBM Corp., Armonk, NY, USA). Descriptive statistics—frequencies, percentages, means, and standard deviations—were used to summarize participant characteristics and questionnaire responses. Associations between sociodemographic variables and knowledge level were assessed by a chi-square test. Multivariable logistic regression was used to identify independent predictors of high knowledge; participants younger than 20 years were combined with the 20–30 year group for the chi-square analysis and excluded from the regression, because the small size of this subgroup could yield unstable estimates. Statistical significance was set at *P* < 0.05.

## RESULTS

A total of 487 participants were included in the study. As Table 1 shows, the majority were female (298, 61.2%) and Saudi nationals (472, 96.9%). Most participants were aged 20–30 years (226, 46.4%), followed by those aged 31–40 (101, 20.7%), 41–50 (92, 18.9%), under 20 (39, 8.0%), and 51 years and over (29, 6.0%). In terms of education, 390 (80.1%) held a bachelor’s degree, 71 (14.6%) had a high school education or less, and 26 (5.3%) held a master’s degree or higher. Monthly family income was relatively balanced across the four bands, ranging from 112 (23.0%) earning 5000–10,000 SAR to 131 (26.9%) earning 10,001–15,000 SAR.

**Table 1:** Participant demographics (*N* = 487)

Demographic variable	Category	*n*	%
Gender	Female	298	61.2
	Male	189	38.8
Nationality	Non-Saudi	15	3.1
	Saudi	472	96.9
Age group (years)	Under 20	39	8.0
	20–30	226	46.4
	31–40	101	20.7
	41–50	92	18.9
	51 and over	29	6.0
Education level	High school or less	71	14.6
	Bachelor’s	390	80.1
	Master’s or higher	26	5.3
Marital status	Single	239	49.1
	Married	248	50.9
Monthly family income	Less than 5000 SAR	118	24.2
	5000–10,000 SAR	112	23.0
	10,001–15,000 SAR	131	26.9
	More than 15,000 SAR	126	25.9

Data are *n* (%). Percentages are of the total sample and sum to 100% within each variable. *N*, total number of participants; *n*, number of participants; SAR, Saudi riyal.

Regarding awareness of G6PD deficiency, Table 2 indicates that 311 respondents (63.9%) had heard of the condition. Furthermore, 288 (59.1%) identified it as a hereditary anemia, and 280 (57.5%) understood it to be a genetic disorder. However, only 207 (42.5%) recognized the parental inheritance pattern. Misconceptions were also evident: 70 (14.4%) thought it was more prevalent in women, 102 (20.9%) believed all patients were always symptomatic, 166 (34.1%) thought the risk was limited to legumes, and 36 (7.4%) incorrectly believed the condition was contagious.

**Table 2:** General awareness of G6PD deficiency (*N* = 487)

Awareness indicator	*n*	%
Heard of G6PD deficiency	311	63.9
Family member with G6PD deficiency	72	14.8
Recognize G6PD deficiency as hereditary anemia	288	59.1
Knowledge that G6PD deficiency is a genetic disorder	280	57.5
Knowledge of parental inheritance pattern	207	42.5
Belief that gender is associated with G6PD deficiency	118	24.2
Belief that G6PD deficiency is more prevalent in females	70	14.4
Awareness that fava beans can trigger hemolytic crisis	290	59.5
Belief that all G6PD-deficient patients are always symptomatic	102	20.9
Belief that risks are limited to legume consumption	166	34.1
Belief that G6PD deficiency is contagious	36	7.4

Values are *n* (%) of respondents answering “Yes”. Percentages are of the total sample. G6PD, glucose-6-phosphate dehydrogenase; *N*, total number of participants; *n*, number of participants.

As shown in Table 3, symptom awareness was variable. While 275 respondents (56.5%) correctly recognized pallor as a symptom of hemolytic crisis and 259 (53.2%) identified jaundice, misconceptions were again present. Notably, 183 (37.6%) believed G6PD deficiency always causes chronic anemia, and 173 (35.5%) incorrectly linked hepatosplenomegaly to the condition.

**Table 3:** Symptom awareness of G6PD deficiency (*N* = 487)

Symptom indicator	*n*	%
Nausea, vomiting, anorexia or diarrhea as severe-crisis symptoms	229	47.0
Jaundice (skin or eye yellowing) as a crisis symptom	259	53.2
Belief that G6PD deficiency always causes chronic anemia	183	37.6
Pallor as a crisis symptom	275	56.5
Hepatosplenomegaly (liver or spleen enlargement) as a crisis symptom	173	35.5
Dizziness as a crisis symptom	226	46.4

Values are *n* (%) of respondents answering “Yes”. Percentages are of the total sample. G6PD, glucose-6-phosphate dehydrogenase; *N*, total number of participants; *n*, number of participants.

Knowledge of triggers was also assessed. According to Table 4, the majority of respondents correctly identified fava beans as a trigger (450, 92.4%). However, many also incorrectly included common foods such as falafel (321, 65.9%) and nuts (308, 63.2%). Only 66 (13.6%) identified aspirin as a trigger, and even fewer selected paracetamol (64, 13.1%) or henna use (64, 13.1%). Misbeliefs were present as well: 59 (12.1%) thought iron supplements could trigger symptoms.

**Table 4:** Knowledge of triggers of hemolysis in G6PD deficiency (*N* = 487)

Trigger	*n*	%
Fava beans	450	92.4
Legumes	370	76.0
Falafel	321	65.9
Nuts	308	63.2
Bacterial or viral infection	139	28.5
Aspirin	66	13.6
Paracetamol	64	13.1
Henna use	64	13.1
Iron supplements	59	12.1

Values are *n* (%) of respondents selecting each trigger. Respondents could select more than one trigger, so categories are not mutually exclusive and percentages do not sum to 100%. G6PD, glucose-6-phosphate dehydrogenase; *N*, total number of participants; *n*, number of participants.

Table 5 presents respondents’ knowledge of appropriate management actions. Most participants reported that avoiding fava beans and certain medications was essential (428, 87.9%), and 353 (72.5%) emphasized the importance of family awareness of triggers. Regular medical check-ups were selected by 351 (72.1%), while 245 (50.3%) supported the use of a medical ID. Still, 77 (15.8%) believed antibiotics could be used without a prescription, and 76 (15.6%) believed iron supplements could be taken without medical consultation.

**Table 5:** Knowledge of management actions in G6PD deficiency (*N* = 487)

Management action	*n*	%
Avoid fava beans and certain medications	428	87.9
Family awareness of triggers causing G6PD symptoms	353	72.5
Regular medical check-ups	351	72.1
Wear medical ID bracelet or card	245	50.3
Increase fluid intake during hemolytic crisis	242	49.7
Use antibiotics without prescription*	77	15.8
Take iron supplements without doctor consultation*	76	15.6

Values are *n* (%) of respondents selecting each action. Respondents could select more than one action, so categories are not mutually exclusive and percentages do not sum to 100%. *Denotes a false statement regarding management. G6PD, glucose-6-phosphate dehydrogenase; *N*, total number of participants; *n*, number of participants.

Participants’ total knowledge scores ranged from 3 to 11 out of a maximum possible score of 33, with a mean score of 6.22 ± 1.51. Although the maximum possible score was high, observed scores clustered within a narrow range, reflecting generally low correct response rates across multiple knowledge domains. Based on the score bands, 264 participants (54.2%) were classified as having low knowledge, and 142 (29.2%) and 81 (16.6%) as having medium and high knowledge, respectively (Figure 1).

**Figure 1:**
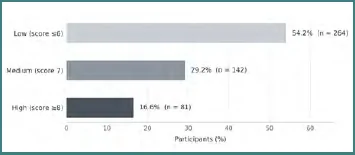
Distribution of participants by knowledge level of G6PD deficiency (N = 487). Knowledge bands were defined from the total score of 33 items: low, score ≤6; medium, score 7; high, score ≥8. Bars show the percentage of participants in each band, with counts in parentheses. G6PD, glucose-6-phosphate dehydrogenase; N, total number of participants; n, number of participants.

Associations between knowledge levels and demographic characteristics are shown in Table 6. A statistically significant association was observed between gender and knowledge level (χ^2^ = 10.577, *P* = 0.005), with a lower proportion of females than males classified in the low-knowledge category (48.7% versus 63.0%). Age was also significantly associated with knowledge level (χ^2^ = 17.541, *P* = 0.008), with participants aged 30 years and under demonstrating the highest proportion of high knowledge. No statistically significant associations were observed between knowledge level and education level or nationality.

**Table 6:** Knowledge level by demographic characteristics (*N* = 487)

Variable	Low, *n* (%)	Medium, *n* (%)	High, *n* (%)	χ^2^	df	*P*
Gender				10.577	2	0.005
Female	145 (48.7)	101 (33.9)	52 (17.4)			
Male	119 (63.0)	41 (21.7)	29 (15.3)			
Age group (years)				17.541	6	0.008
30 and under	129 (48.7)	77 (29.1)	59 (22.3)			
31–40	59 (58.4)	35 (34.7)	7 (6.9)			
41–50	57 (62.0)	23 (25.0)	12 (13.0)			
51 and over	19 (65.5)	7 (24.1)	3 (10.3)			
Education level				1.372	4	0.849
High school or less	40 (56.3)	18 (25.4)	13 (18.3)			
Bachelor’s degree	208 (53.3)	118 (30.3)	64 (16.4)			
Master’s or higher	16 (61.5)	6 (23.1)	4 (15.4)			
Nationality				1.132	2	0.568
Non-Saudi	7 (46.7)	4 (26.7)	4 (26.7)			
Saudi	257 (54.4)	138 (29.2)	77 (16.3)			

Values are *n* (%) of knowledge levels within each demographic category; rows within a variable sum to 100%. Comparisons were made with the χ^2^ test of independence; the χ^2^, df and *P* values are given on the variable row and apply to the variable as a whole, so those cells are intentionally blank on the category rows. Bold *P* values indicate statistical significance (*P* < 0.05). *N*, total number of participants; *n*, number of participants.

Multivariable logistic regression analysis was conducted to identify independent predictors of high knowledge (Table 7). High knowledge was used as the outcome variable, with low and medium knowledge combined as the reference category. Participants younger than 20 years were excluded from the regression analysis due to the small sample size in this subgroup. After adjustment for covariates, age was the only variable independently associated with high knowledge. Participants aged 31–40 years had significantly lower odds of high knowledge compared with those aged 20–30 years (OR = 0.260; 95% CI, 0.114–0.591; *P* = 0.002). Although participants aged 41–50 years and those aged 51 years and older also showed lower odds of high knowledge, these associations did not reach statistical significance. Gender, nationality, and educational level were not significant independent predictors of high knowledge.

**Table 7:** Multivariable logistic regression predicting high knowledge of G6PD deficiency (*N* = 487)

Predictor	OR	95% CI	*P*
Age 31–40 vs 20–30 years	0.260	0.114–0.591	0.002
Age 41–50 vs 20–30 years	0.524	0.267–1.026	0.058
Age 51 and over vs 20–30 years	0.403	0.118–1.378	0.190
Male vs female	0.857	0.522–1.408	0.629
Saudi vs non-Saudi	0.536	0.166–1.727	0.479
Bachelor’s vs secondary or less	0.876	0.453–1.692	0.911
Master’s or higher vs secondary or less	0.811	0.239–2.757	0.911

Outcome: high knowledge versus low or medium knowledge combined. Estimates are from a multivariable binary logistic regression model containing age, gender, nationality, and education level. Reference categories: age 20–30 years, female gender, non-Saudi nationality, secondary education or less. Participants younger than 20 years (*n* = 39) were excluded from the regression analysis. Bold *P* values indicate statistical significance (*P* < 0.05). CI, confidence interval; G6PD, glucose-6-phosphate dehydrogenase; *N*, total number of participants; *n*, number of participants; OR, odds ratio.

## DISCUSSION

G6PD deficiency is the most common enzyme deficiency affecting red blood cells globally and has a high prevalence in Saudi Arabia [10]. Our study assessed the awareness of residents in the Al-Baha region and found a generally low level of knowledge.

Although 63.9% of participants had heard of G6PD deficiency, the overall knowledge level was low. In contrast, a study in Jazan reported fair knowledge [12]. This difference may reflect methodological differences between the two surveys, including the proportion of participants with a family member affected by G6PD deficiency (14.8% in our study versus 16.8% in Jazan).

Knowledge levels were higher among females than males, consistent with findings from Jazan and the Eastern Province [12,15]. Participants aged 30 years and under had the highest proportion with high knowledge (22.3%), similar to Jazan, where individuals aged 20–40 years had the highest knowledge [12]. This may reflect younger participants’ greater access to technology and online health information, whereas older individuals may face more difficulty using these resources.

In the multivariable analysis, age was the only independent predictor of high knowledge, with participants aged 31–40 years showing significantly lower odds than those aged 20–30 years. Gender, nationality, and education level were not independent predictors, indicating that the gender association observed in the unadjusted analysis did not persist after adjustment.

Educational level was not associated with awareness in our study, aligning with findings from the Eastern Province [15] but differing from Jazan [12]. This suggests that G6PD deficiency is not consistently included in educational curricula or public health messaging.

General awareness of the condition was moderate, but understanding of inheritance, symptoms, and triggers was limited. Most participants correctly identified fava beans (92.4%) and legumes (76.0%) as triggers, while knowledge of falafel (65.9%) and nuts (63.2%) was fair. Awareness of non-dietary and drug-related triggers was low, including infections (28.5%), aspirin (13.6%), paracetamol (13.1%), and henna (13.1%). These findings are consistent with studies conducted in Jeddah and other regions of Saudi Arabia [14,16]. Limited awareness of these triggers can lead to preventable hemolytic crises, highlighting the need for urgent health education strategies.

Most participants recognized appropriate management strategies, such as avoiding triggers (87.9%), promoting family awareness (72.5%), attending regular medical check-ups (72.1%), and wearing a medical ID (50.3%). These results align with Al Blewi *et al*. [9]. However, misconceptions persisted, with some participants believing that using antibiotics without prescription (15.8%) or taking iron supplements without medical advice (15.6%) could improve G6PD deficiency, emphasizing the need for targeted educational interventions.

The study has several strengths. It included a relatively large sample (*n* = 487) with diverse demographic representation and used a structured questionnaire adapted from previously published instruments, reviewed by a hematology consultant and pilot tested before distribution. Online distribution allowed wide regional coverage.

Several limitations should also be considered. Voluntary online participation may have introduced selection bias toward individuals with greater internet access and interest in health topics, which may limit the generalizability of the findings. The exclusion of healthcare professionals may further affect generalizability, and the use of self-reported data may be subject to reporting bias. Additionally, the cross-sectional design limits causal inference.

Overall, our findings highlight a serious gap in essential knowledge about G6PD deficiency that can have preventable, potentially fatal consequences. Community-based awareness programs, school health education, and physician-led counseling are urgently needed to increase awareness, improve preventive measures, and reduce avoidable complications associated with G6PD deficiency.

## CONCLUSION

This study highlights a considerable gap in comprehensive knowledge of G6PD deficiency among the general population in the Al-Baha region. While basic awareness of the condition is present, especially regarding common dietary triggers such as fava beans, knowledge of other critical aspects—drug-induced hemolysis, symptom recognition, and proper management practices—remains insufficient. Younger age groups showed better understanding, pointing to a generational divide in health education. These findings underscore the urgent need for targeted educational interventions and awareness campaigns to enhance community understanding and prevent avoidable complications associated with G6PD deficiency. Enhancing public awareness will not only reduce the health burden but also empower individuals and families to take proactive preventive measures.

## Data Availability

The data supporting the findings of this study are available from the corresponding author upon reasonable request.
